# Development of Polysorbate 80/Phospholipid mixed micellar formation for docetaxel and assessment of its *in vivo *distribution in animal models

**DOI:** 10.1186/1556-276X-6-354

**Published:** 2011-04-20

**Authors:** Hua Song, Hongquan Geng, Jing Ruan, Kan Wang, Chenchen Bao, Juan Wang, Xia Peng, Xueqing Zhang, Daxiang Cui

**Affiliations:** 1 National Key Laboratory of Nano/Micro Fabrication Technology, Key Laboratory for Thin Film and Microfabrication of Ministry of Education, Department of Bio-Nano Science and Engineering, Institute of Micro-Nano Science and Technology, Shanghai Jiao Tong University, 800 Dongchuan Road, Shanghai 200240, People's Republic of China; 2 Department of Pediatric Urology, Xinhua Hospital, Shanghai Jiao Tong University School of Medicine, Shanghai 200092, People's Republic of China; 3 Department of Laboratory Medicine, Shanghai First People's Hospital, Shanghai Jiao Tong University, 85 Wujin Road, Shanghai 200080, People's Republic of China

## Abstract

Docetaxel (DTX) is a very important member of taxoid family. Despite several alternative delivery systems reported recently, DTX formulated by Polysorbate 80 and alcohol (Taxotere^®^) is still the most frequent administration in clinical practice. In this study, we incorporated DTX into Polysorbate 80/Phospholipid mixed micelles and compared its structural characteristics, pharmacokinetics, biodistribution, and blood compatibility with its conventional counterparts. Results showed that the mixed micelles loaded DTX possessed a mean size of approximately 13 nm with narrow size distribution and a rod-like micelle shape. In the pharmacokinetics assessment, there was no significant difference between the two preparations (*P *> 0.05), which demonstrated that the DTX in the two preparations may share a similar pharmacokinetic process. However, the Polysorbate 80/Phospholipid mixed micelles can increase the drug residence amount of DTX in kidney, spleen, ovary and uterus, heart, and liver. The blood compatibility assessment study revealed that the mixed micelles were safe for intravenous injection. In conclusion, Polysorbate 80/Phospholipid mixed micelle is safe, can improve the tumor therapeutic effects of DTX in the chosen organs, and may be a potential alternative dosage form for clinical intravenous administration of DTX.

## Introduction

As the most successful chemotherapeutic drugs currently available, Taxanes play an important role in the treatment of various solid tumors [1]. As a second-generation semi-synthetic taxane derivative, docetaxel (DTX) is about twice as potent as paclitaxel in inhibiting microtubule depolymerization, and has the unique ability to alter certain classes of microtubules [2], which differs from most spindle poisons currently used in clinic. However, the clinical intravenous administration of commercially available DTX (Taxotere^®^) is formulated in a highly concentrated solution containing 40-mg DTX and 1040-mg Tween^® ^80 (Polysorbate 80) per mL. This concentrated solution has to be carefully diluted with solvent containing 13% ethanol in saline before administration, and has to be used within 4 h for its limited stability. These shortcomings bring great inconvenience to the practical application.

As a result, current research is mainly focused on developing new preparations of DTX to improve the therapeutic index and reduce the adverse reactions [3]. Various drug delivery systems have been reported recently, such as DTX loaded nanoparticles [4], liposomes [5], *N*-palmitoyl chitosan anchored DTX liposomes [6], self-emulsified DTX [7], PEGylated liposomes [8], PEGylated immunoliposomes [9], and PEG-liposomes-folic acid bioconjugates [10]. Although they have their advantages, respectively, each of the above is hampered by one or more problems, such as complicated preparation process, high cost, and low stability of the formulation. Therefore, Taxotere^® ^is still the most widely used clinical DTX preparation currently available. Increasing evidence highly suggest that when drug is incorporated into different carriers, its pharmacokinetics may be completely altered. Therefore, there is an urgent need for a pharmaceutical composition comprising DTX, which should have high solubility and stability, simplified preparation process, and the same pharmacokinetics as Taxotere^®^.

As well known, Phospholipid is an important structural component of cell membranes and a biocompatible material with an excellent biocompatibility [11]. The physical and chemical properties of Polysorbate/Phospholipid mixed aggregates have been report previously [12]. In our previous report, we optimized the conditions to prepare DTX-loaded Polysorbate 80/Phospholipid mixed micelles based on the preparation of Taxotere^®^, revealed the efficient and stable encapsulation of DTX in the mixed micelles by using the self-assembly method [13,14], and demonstrated the mixed micelles can prolong the stable time of DTX injection to 3 days. The primary aim of the present study is to further evaluate the characteristics of the DTX-loaded mixed micelles, and pharmacokinetics, tissue distribution, and blood compatibility. This may provide new idea for solving the problems faced by Taxotere^® ^such as the complicated steps of clinical preparation, inaccurate dosage, and low stability of the preparation.

## Experimental

### Materials

Phospholipid was provided by Taiwei Pharmaceutical Factory (Shanghai, China, *M*n = 760.08). Polysorbate 80 was purchased from Shanghai Shenyu Pharmaceutical & Chemical Co. Ltd (Shanghai, China, *M*n = 1309.65). DTX (99.4%) was obtained from Shanghai Junjie Bio-Engineering Co. Ltd. (Shanghai, China, *M*n = 807.88). Heparinsodium injection, 10,000 IU/mL was purchased from Shanghai No. 1 Biochemical Pharmaceutical Co. Ltd. (Shanghai, China). Glucose solution 5% was obtained from Shanghai Baite Medical Product Co. Ltd. (Shanghai, China). Spectra-grade reagents were used as the mobile phase in high-performance liquid chromatography (HPLC) analysis, and all other reagents were analytical grade and used without further purification. Distilled and deionized water was used in all experiments.

### Animals

Male Sprague-Dawley (SD, 8 weeks old, 200 ± 20 g) rats and female Kunming strain mice (8 weeks old, 20 ± 2 g) were obtained from Second Military Medical University of Chinese PLA. All the pathogen-free animals were acclimatized at a temperature of 25 ± 2°C and a relative humidity of 70 ± 5% under natural light/dark conditions for at least 24 h before dosing. The experiments were carried out in compliance with the National Institute of Health Guide for the Care and Use of Laboratory Animals.

### Preparation of DTX-loaded Polysorbate 80/Phospholipid mixed micelles

DTX-loaded Polysorbate 80/Phospholipid mixed micelles were prepared by means of the self-assembly method as we described previously [15]. Briefly, the response surface methodology was used to optimize the preparation of the mixed micelles: DTX (5 mg), Polysorbate 80 (125 μL), and Phospholipid (30 mg) were dissolved in 0.3 mL of dehydrated ethanol with the help of stirring at room temperature, then the homogeneous phase was injected rapidly into the 5% glucose solution in order to obtain a clear mixed micelle solution with a final volume of 10 mL, then the mixed micelles solution was filtrated through a 0.22-μm pore-sized membrane for further investigation. The final concentration of DTX is 0.62 mM, Polysorbate 80 is 10.16 mM, and Phospholipid is 3.95 mM.

The mean diameter and particle size distribution of the DTX-loaded Polysorbate 80/Phospholipid mixed micelles were determined by a particle-size analyzer (Mastersizer 2000, Malvern Instruments, Ltd., Malvern, U.K.), based on the laser dynamic light scattering technique. Sample solutions filtered through a 0.22-μm filter membrane were transferred into the light scattering cells. The intensity autocorrelation was measured at a scattering angle of 90° at room temperature. The morphological examination of micelles was performed using a JEOL JEM-2010 transmission electron microscope (TEM) at an acceleration voltage of 120 kV. In practice, one drop of solution with the sample was placed on a 400-meshes copper grid precoated with a carbon film and allowed to dry further for 30 min, then examined with the electron microscope.

An aliquot of DTX-loaded Polysorbate 80/Phospholipid mixed micelles was treated with four times volume of dehydrated ethanol to disrupt the micelle structure. Level of encapsulated DTX was measured using a reverse phase HPLC method. Stock solutions of DTX (0.2 mg/mL) were prepared by dissolving 10 mg of DTX in 10 mL dehydrated ethanol, followed by addition of 40 mL distilled water, and standard curve was set up with satisfactory linearity. The HPLC system consisted of a HP HPLC (3D) series equipped with G1322A online degasser and G1311A quaternary pump (Agilent Technologies, Palo Alto, CA, USA) was used. Chromatographic separations were achieved using a Diamonsil C18 column (5 μm, 250 × 4.6 mm, Dikma Technologies Inc, Lake Forest, CA, USA) at 25C. The mobile phase consisted of deionized water and HPLC grade acetonitrile [45:55 (*V*/*V*)]. The samples were delivered at a flow rate of 1 mL/min and detected at 230 nm using G1314A VWD detector (Agilent Technologies, Palo Alto, CA, USA).

### Pharmacokinetic study

Sprague-Dawley rats were used to examine the pharmacokinetics of DTX encapsulated in Polysorbate 80/Phospholipid mixed micelles. Rats were randomly divided into following two groups (*n *= 6, half male and half female): (1) DTX encapsulated in Polysorbate 80 micelles (75 mg/m^2^); (2) DTX encapsulated in Polysorbate 80/Phospholipid mixed micelles (75 mg/m^2^). Drugs were intravenously administrated trough the tail vein. The blood samples (0.5 mL) were collected into heparinized tubes via the femoral vein at 5, 15, 30 min, 1, 2, 4, 6, 8, and 12 h. The plasma was obtained by centrifugations at 900 × *g *for 10 min. Plasma samples were frozen and maintained at -20°C before analysis.

### Tissue biodistribution study

To assess the effect of Polysorbate 80/Phospholipid mixed micelle formulation of DTX on tissue distribution, 72 female Kunming strain mice were randomly divided into two groups. The administration protocol of tissue distribution study was as same as that used in the pharmacokinetics. At 5, 15, 30 min, 1, 2, 3, 4, 6, and 8 h after drug injection, each animal (*n *= 4 for each time point) was euthanized and heart, spleen lung, liver, kidney, uterus and ovaries, brain as well as blood samples were collected. Tissue samples were blotted with paper towel, rinsed in ice-cold saline, blotted to remove excess fluid, weighed, and stored at -50°C until required for analysis.

Aliquots of 0.1 g tissue samples were minced into small pieces (1 mm^3 ^on average), homogenized in the mixed solution of acetonitrile and water (50:50, *V*/*V*) with a ultrasonic Cell Disrupter System (JY92-, Ningbo Scientz Biotechnology Co., Ltd, Ningbao, China), and vortexed for 1 min. After centrifugation at 15000 × *g *for 3 min, the 100-μL clear supernatant was removed and extracted by 600-μL *tert*-butyl methyl ether, the organic phase was separated and evaporated under a gentle stream of nitrogen. Then, the residue was dissolved in 40 μL of acetonitrile, centrifuged at 1400 × *g *for 5 min, and aliquots of 20 μL were injected into the HPLC system. The concentrations of DTX in tissue samples were determined by the HPLC method as same as the analysis of the mice plasma samples.

### Serum sample analysis

DTX levels in plasma and tissue were measured by reverse-phase HPLC method. Briefly, 200 μL of plasma was extracted twice with 200-μL *tert*-butyl methyl ether. The total clear organic layer was separated by centrifugation at 15000 × *g *for 3 min, and evaporated under a gentle stream of nitrogen. The residue was then dissolved by 40 μL acetonitrile, centrifuged at 1400 × *g *for 5 min, and aliquots of 20 μL were injected into the HPLC system. The rat plasma samples employed the mobile phase consisted of HPLC grade acetonitrile, deionized water, tetrahydrofuran, ammonium hydroxide solution (25%), and acetic acid solution (36%) [55**:**45**:**3**:**0.03**:**0.06 (*V*/*V*)]; the mice plasma samples employed the mobile phase consisted of HPLC grade acetonitrile, deionized water, and tetrahydrofuran [55**:**45**:**4 (*V*/*V*)]. All the analyses with a flow rate of 1.0 mL/min for the mobile phase, the retention time of DTX in rat plasma samples and mice plasma samples were approximately 10.7 and 11.2 min, respectively.

### Hemolysis test

Rat blood was used to test the hemolysis effect of DTX-loaded Polysorbate 80/Phospholipid mixed micelles. Briefly, the fibrinogen was removed from 10 mL of rat blood by stirring the blood with glass rod. Ten milliliters of 5% glucose injection solution was added into defibrinogen blood sample, and supernatant was removed after centrifugation at 900 × *g *for 10 min. The erythrocyte pellets at the bottom of centrifuge tube were washed for four times (centrifugation followed by re-dispersion) with 5% glucose injection solution. Finally, after repeated washing and centrifugation, an adequate amount of 5% glucose injection was added to the erythrocyte pellets to give a 2% erythrocyte standard dispersion and stored at 4°C for further use. The DTX-loaded Polysorbate 80 micelles (Taxotere^®^), and DTX-loaded Polysorbate 80/Phospholipid mixed micelles were dispersed in 5% glucose injection solution with DTX concentration of 0.5 mg/mL and Polysorbate 80 of 1.25%, respectively. The different amounts of micelle solution with volume of 0.1, 0.2, 0.3, 0.4, and 0.5 mL (NO. 1, 2, 3, 4, and 5) were added into six tubes with 2.5 mL of 2% erythrocyte dispersion in each. Then adequate amounts of 5% glucose injection solution were added in every tube to obtain a final volume of 5 mL. Negative control (NO. 6) was 5% glucose injection, and positive control (NO. 7) was prepared by adding 2.5 mL of ultra- pure water into 2.5 mL of 2% erythrocyte dispersion instead of 5% glucose injection and micelle solution. After vortexing, the tubes were incubated at 37°C and observed microscopically from 15 min to 1 h. Then, the tubes were centrifuged at 900 × *g *for 10 min. At last, the optical density (OD) was obtained from a fast wavelength scanning between 200 and 1100 nm by a UV-spectrophotometer (11000, Beijing analysis Instrument Co., Ltd, Beijing, China) at 418 nm. The hemolysis ratio (HR) was calculated according to the equation: HR = [(OD_t _- OD_n_)/(OD_p _- OD_n_)] × 100%. Here, the OD_t _means the OD value of tested group, the OD_n _and OD_p _were OD value of negative and positive control, respectively.

### Plasma protein binding test

Equilibrium retrodialysis was used to evaluate the plasma protein binding ability of DTX captured in Polysorbate 80 micelles and DTX captured in Polysorbate 80/Phospholipid mixed micelles, respectively. Firstly, 4-mL rat plasma was full mixed with 1.2 mL micelles solution for 0.5 h in 10 mL sealed glass cells with 120 rpm magnetic stirring at 36.5 ± 0.5°C, then precise ultra- pure water was added into the mixed solution for making up the loss of weight; secondly, dialysis bags (cellulosic membranes with molecular weight cut-offs of 10000 Da; Millipore, USA) filled with 0.5 mL 5% glucose injection were put into the sealed cells and the retrodialysis was carried out for 6 h at 36.5 ± 0.5°C with 120 rpm magnetic stirring in temperature controlled water bath. After cooling to the ambient temperature and making up the loss of weight with ultra-pure water, aliquots of 200 μL plasma and resulting dialysate were promptly recovered from the glass cells and analyzed by HPLC method mentioned above, respectively. The percentage of plasma protein binding rate of DTX (*B*%) was calculated as: . The *C*_o _and *C*_i _were the drug concentrations in the plasma outside the dialysis bags and the dialysate inside the dialysis bags (ng/mL). 0.5 and 5.2 means the volume of the solution inside and outside the dialysis bags respectively (mL).

### Statistical analysis

The compartment of model was simulated by 3p87 program (Practical Pharmacokinetic Program, 1987, China) and the parameters of pharmacokinetics were obtained. The calculation of AUC was based on statistical moment theory. The pharmacokinetic parameters were analyzed for statistical significance by unpaired Student's *t-*test. For this purpose, the level of significance was set at *α *< 0.05. In the tissue distribution studies, the AUC could not be determined in individual mice because of the destructive study design.

## Results

### Characterization of DTX-loaded Polysorbate 80/Phospholipid mixed micelles

**Figure 1:**
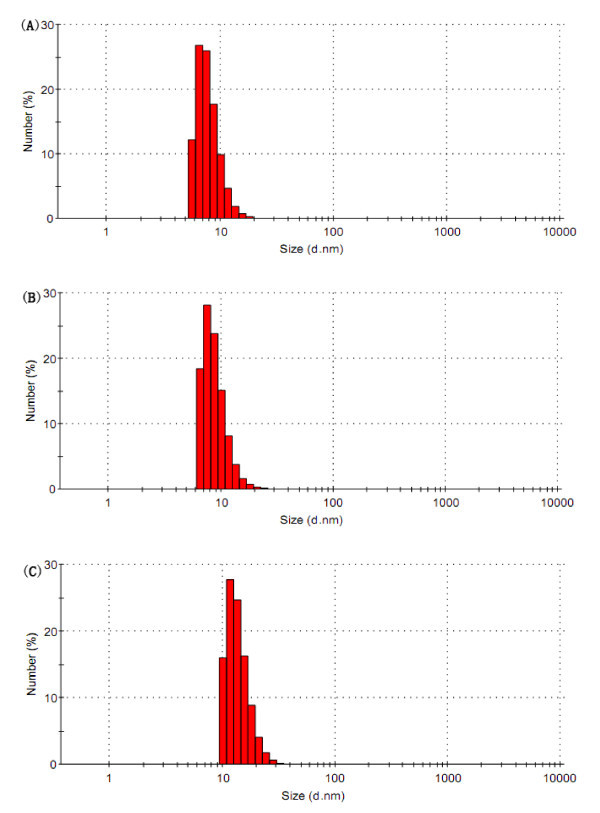
**Particle size distributions of the micelles**. **(A) **DTX-loaded Polysorbate 80 micelles; **(B) **Blank Polysorbate 80/Phospholipid mixed micelles; **(C) **DTX-loaded Polysorbate 80/Phospholipid mixed micelles.

**Figure 2:**
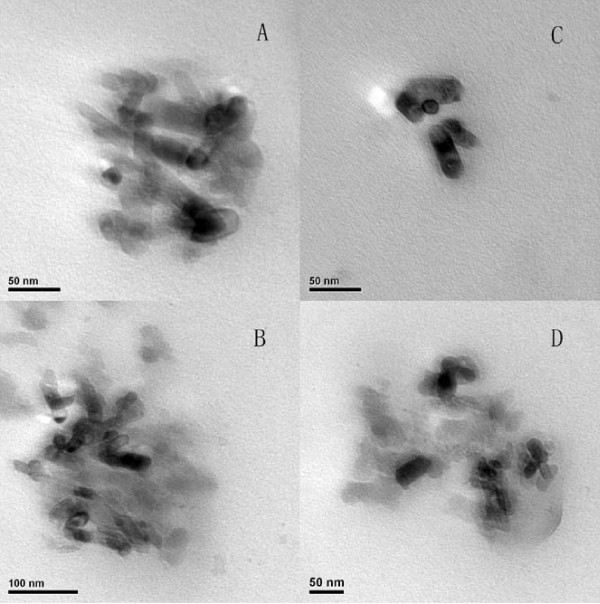
**Transmission electron microscope (TEM) photograph of DTX-loaded Polysorbate 80/Phospholipid mixed micelles (A, B, C) and blank Polysorbate 80/Phospholipid mixed micelles (D)**.

In order to achieve longevity during systemic circulation, the micelles must be small enough to evade detection and destruction of the reticuloendothelial system (RES). The mean diameter and the polydispersity coefficient (PDI) of DTX-loaded Polysorbate 80 micelles, blank Polysorbate 80/Phospholipid mixed micelles and DTX-loaded Polysorbate 80/Phospholipid mixed micelles were 7.89 ± 1.97 nm and 0.234, 8.44 ± 2.34 nm and 0.319, 13.89 ± 3.52 nm and 0.089, respectively, which were measured by dynamic light scattering. It could be seen that the size distribution was relatively narrow (Figure 1). The hydrodynamic particle size of the drug-loaded micelles is understandably larger than the blank micelles, probably due to the incorporation of large and bulky drug molecules (*M*w of DTX: 807.88 g/mol) within the core. Moreover, the particle size of the drug-loaded mixed micelles is larger than drug-loaded Polysorbate 80 single component micelles, it is still safely below 20 nm. There was no significant difference in particle size of these three types of micelles. The DTX-loaded Polysorbate 80/Phospholipid mixed micelles were dispersed in pure water and the morphology was investigated by TEM. These drug-loaded particles had a rod-like shape, which is one of the characteristic shapes of micelle. The particle surface was very smooth and no drug crystal was visible (Figure 2).

### Pharmacokinetic study

**Figure 3:**
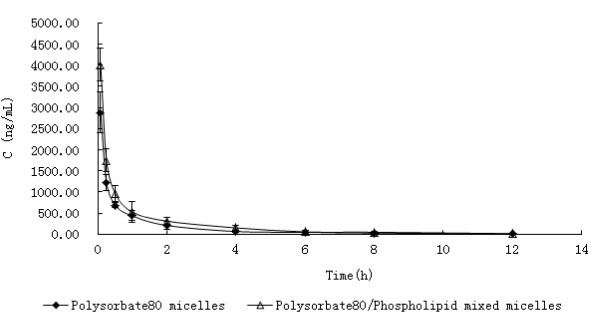
**Mean plasma concentration-time profiles of DTX after *i.v*. administration of a single 75 mg/m^2 ^dose of DTX-loaded Polysorbate 80 micelles and DTX-loaded Polysorbate 80/Phospholipid mixed micelles to rats (Each point represents the mean ± SD of six rats.)**.

The drug concentration of rats' plasma was detected by HPLC analysis, which has been validated with a linear calibration curve in the range of 10 to 6000 ng/mL of DTX and the correlation coefficient over this concentration range was 0.9996. The plasma concentration time profiles of DTX in rat plasma after intravenous administration of DTX-loaded Polysorbate 80 micelles and DTX-loaded Polysorbate 80/Phospholipid mixed micelles at a single dose of 75 mg/m^2 ^were compared (Figure 3). The profiles showed a rapid decline in the first 1 h (distribution phase) after dosing the two preparations. Furthermore, the DTX concentration versus time plots was obtained by means of a three compartmental model with the weight coefficient of 1/*C*^2 ^based on computer program 3p87. The pharmacokinetic parameters are shown in Table 1 and analyzed for statistical significance by unpaired Student's *t*-test. The results of the statistical analysis proved the pharmacokinetic parameters were no significant difference (*P *> 0.05) between the two preparations. These data demonstrated that the DTX in Polysorbate 80 micelles and the DTX in Polysorbate 80/Phospholipid mixed micelles can achieve a similar pharmacokinetic process in rats.

**Table 1:** Pharmacokinetic parameters of DTX after *i.v*. administration of DTX-loaded Polysorbate 80 micelles and DTX-loaded Polysorbate 80/Phospholipid mixed micelles to rats.

Parameters	Unit	DTX-loaded Polysorbate 80 micelles	DTX-loaded Polysorbate 80/Phospholipid mixed micelles	*P*
*t*_1/2α_	h	0.916 ± 0.608	0.919 ± 0.601	0.995
*t*_1/2β_	h	4.901 ± 2.453	6.726 ± 1.684	0.626
*K*_12_	h^-1^	4.780 ± 2.525	2.886 ± 1.551	0.179
*K*_21_	h^-1^	2.544 ± 1.187	2.376 ± 1.421	0.828
*K*_13_	h^-1^	0.773 ± 0.581	2.350 ± 1.577	0.095
*K*_31_	h^-1^	0.190 ± 0.122	0.376 ± 0.404	0.310
*K*_10_	h^-1^	2.800 ± 0.897	2.735 ± 0.891	0.908
AUC_0-12 h_	μg∙L^-1^∙h	2024.891 ± 287.679	2218.685 ± 407.753	0.379
Cl	L∙h	0.038 ± 0.006	0.038 ± 0.007	0.468

*n *= 6.

Mean ± SD.

**Figure 4:**
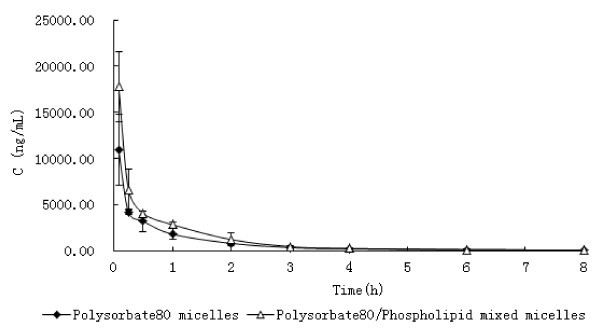
**Mean plasma concentration-time profiles of DTX after *i.v*. administration of a single 75 mg/m^2 ^dose of DTX-loaded Polysorbate 80 micelles and DTX-loaded Polysorbate 80/Phospholipid mixed micelles to mice (Each point represents the mean ± SD of four mice.)**.

The mice's plasma drug concentration was detected by HPLC analysis, the calibration curve having DTX concentrations ranging from 50 to 30000 ng/mL for plasma exhibited good linearity, and the correlation coefficient over this concentration range was 0.9999. Results showed that the plasma DTX concentration-time profiles observed in mice after intravenous administration of DTX-loaded Polysorbate 80 micelles and DTX-loaded Polysorbate 80/Phospholipid mixed micelles at a single dose of 75 mg/m^2 ^were similar to the pharmacokinetic study in rats. The time of distribution phase was short and the concentration decreased quickly in this phase (Figure 4). The pharmacokinetic parameters are shown in Table 2 and analyzed for statistical significance. Results showed all pharmacokinetic parameters were no significant difference (*P *> 0.05) except *K*_13_. The result suggested that the DTX in two preparations can achieve a similar pharmacokinetic process in mice. This was in consistent with the result of the intravenous administration in rats.

**Table 2:** Pharmacokinetic parameters of DTX after *i.v*. administration of DTX-loaded Polysorbate 80 micelles and DTX-loaded Polysorbate 80/Phospholipid mixed micelles to mice.

Parameters	Unit	DTX-loaded Polysorbate80 micelles	DTX-loadedPolysorbate 80/Phospholipid mixed micelles	*P*
*t*_1/2α_	h	0.682 ± 0.2088	0.634 ± 0.166	0.734
*t*_1/2β_	h	15.790 ± 20.5700	5.695 ± 1.703	0.445
*K*_12_	h^-1^	13.976 ± 7.9457	9.627 ± 6.878	0.440
*K*_21_	h^-1^	2.198 ± 0.2320	2.739 ± 1.032	0.346
*K*_13_	h^-1^	4.410 ± 1.4573	1.012 ± 0.687	0.022*
*K*_31_	h^-1^	0.149 ± 0.108	0.155 ± 0.042	0.927
*K*_10_	h^-1^	16.040 ± 12.268	14.245 ± 16.245	0.866
AUC_0-8 h_	μg∙L^-1 ^∙h	10466.290 ± 3122.821	11406.914 ± 5042.349	0.762
Cl	L∙h	0.009 ± 0.007	0.008 ± 0.003	0.776

*n *= 4. Mean ± SD.

* *P *< 0.05, denotes significant difference between two groups.

### Tissue distribution study

The tissue distribution profiles of DTX after intravenous administration of DTX-loaded Polysorbate 80/Phospholipid mixed micelles to mice was investigated with DTX encapsulated in Polysorbate 80 micelles as reference. The standard curves of the peak area (*Y*) to the concentration (*C*) for heart, liver, spleen, lung, ovary and uterus, kidney, and brain are listed in Table 3. The calibrations were linear over a certain range in all biosamples with a correlation coefficient (*R*) larger than 0.9990.

**Table 3:** Standard curves, correlation coefficients, and linear ranges of DTX in mice tissue samples.

Biosamples	Standard curves	Correlation coefficients (*R*)	Linear ranges (ng/mL)
Heart	*Y *= 0.0397*C *- 2.0823	0.9997	100-5000
Liver	*Y *= 0.0428*C *- 8.8082	0.9997	100-30000
Spleen	*Y *= 0.0417*C *- 3.6669	0.9996	100-10000
Lung	*Y *= 0.0387*C *- 0.0056	0.9993	100-30000
Ovary and uterus	*Y *= 0.0400*C *- 3.1940	0.9990	100-4000
Kidney	*Y *= 0.0428*C *- 8.9972	0.9996	100-30000
Brain	*Y *= 0.0406*C *- 4.1897	0.9994	100-40000

**Figure 5:**
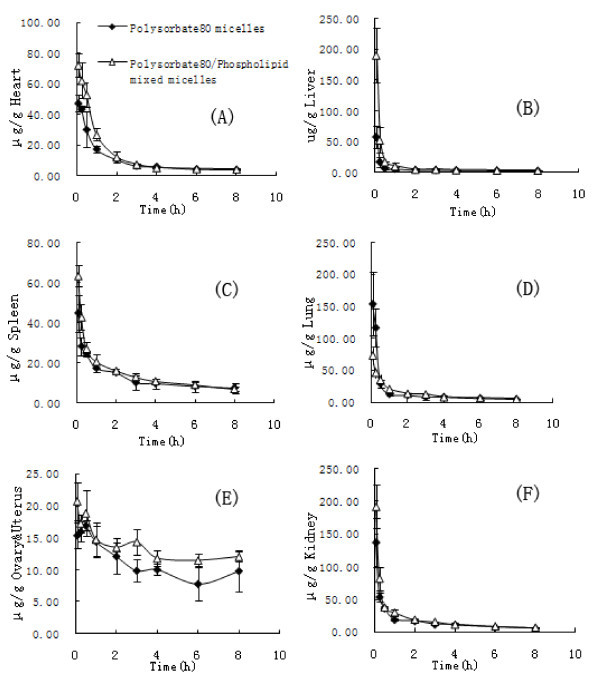
**Mean concentration-time profiles of DTX in (A) heart, (B) liver, (C) spleen, (D) lung, (E) ovary/uterus, and (F) kidney, and following intravenous administration of a single 75 mg/m^2 ^dose of DTX Polysorbate 80 micelles injection and DTX Polysorbate 80/Phospholipid mixed micelles injection to mice (Each point represents the Mean ± SD of four mice**.).

The DTX-AUC (DTX-area under curve) of the two preparations in different tissues, including plasma, heart, spleen, lung, ovary and uterus, kidney, and liver were calculated (Table 4). As shown in Figure 5, DTX was widely and rapidly distributed into most tissues following intravenous administration of the two micelle preparations. The DTX-AUC of Polysorbate 80/Phospholipid mixed micelles was higher in all tissues compared to the reference. The order in DTX-AUC from the highest to the lowest for DTX-loaded Polysorbate 80 micelles was kidney > lung > spleen > ovary and uterus > heart > liver > plasma. In contrast, the corresponding order for the DTX-loaded Polysorbate 80/Phospholipid mixed micelles was kidney > spleen > ovary and uterus > lung > heart > liver > plasma, and the DTX concentration in brain was too low to be detected.

**Table 4:** The AUC^a ^of DTX in mice (*n *= 4) tissues after *i.v*. administration of DTX Polysorbate 80 micelles injection and DTX Polysorbate 80/Phospholipid mixed micelles injection.

Tissues	DTX-loaded Polysorbate 80 micelles(μg·h·g-1)	DTX-loaded Polysorbate 80/Phospholipid mixed micelles(μg·h·g-1)	Ratio b
Plasma	10.466	11.407	1.090
Heart	74.263	99.451	1.339
Liver	42.081	71.695	1.704
Spleen	94.203	110.134	1.169
Lung	102.941	103.642	1.007
Ovary and uterus	84.217	104.152	1.237
Kidney	117.103	144.046	1.230

^a^AUC of the tissues, 0 to 8 h.

^b^The ratio was AUC_Polysorbate 80/Phospholipid mixed micelles_/AUC_Polysorbate80 micelles._

### Hemolysis test

Complete hemolysis was observed in tube of positive control at 15 min. The solution was red clear-diaphanous and there was no erythrocyte detected at the bottom of the tube. The erythrocyte precipitated at the bottom of other six tubes could be dispersed after shaking, and the supernatant was achromatic and transparent in the period of 1 h observation. The optical density test results (Table 5) showed that the hemolysis rate of all the micelles is below 5%, and all the hemolysis ratios of the Polysorbate 80/Phospholipid mixed micelles were lower than Polysorbate 80 micelles. These suggested that all DTX-loaded micelles made from Polysorbate 80 in these concentrations had no destructive effect on erythrocyte, and the DTX-loaded new formulation caused much gentler hematolysis and erythrocyte agglutination at body temperature compared to the marketed DTX-loaded Polysorbate 80 micelles.

**Table 5:** Hemosysis test of DTX-loaded Polysorbate 80 micelles and DTX-loaded Polysorbate 80/Phospholipid mixed micelles.

**No**.	1	2	3	4	5	6	7
HR of DTX-loaded Polysorbate 80 micelles (%)	1.046	1.535	1.839	2.260	3.745	0	100
HR of DTX-loaded Polysorbate 80/Phospholipid mixed micelles	0.928	1.181	1.552	2.075	2.480	0	100
(%)							

Results were shown on mean ± SD (*n *= 3).

### Plasma protein binding test

The plasma protein binding rate of DTX captured in Polysorbate 80 micelle and DTX captured in Polysorbate 80/Phospholipid mixed micelle were 86.05 ± 2.41 and 84.21 ± 2.21% (*n *= 3 repeats per measurement), respectively, which showed that the plasma protein binding rates of the DTX were relatively high in both Polysorbate 80 micelles and Polysorbate 80/Phospholipid mixed micelles. No significant difference existed between them (*P >*0.05) (Table 6).

**Table 6:** The binding efficiencies (%) between DTX and the proteins in plasma (*n *= 3).

No.	Polysorbate80 micelles	Polysorbate80/lecithin mixed micelles
Mean ± SD	84.21 ± 2.21	86.05 ± 2.41

*P*	0.3841	

## Discussion

To our knowledge, very little information has been published about particle properties and solubilization ability of Polysorbates, including Polysorbate 80. In particular, no other detailed study has dealt with the research of pharmocokinetics and tissue distribution of the drug vesicles with Phospholipid and Polysorbate 80. This made the discussion of our present results not only more difficult but also more interesting. We have previously demonstrated the blank Polysorbate 80/Phospholipid mixed micelles has a far lower critical micelle concentration (CMC) values than the blank Polysorbate 80 micelles with pyrene fluorescence probe spectrometry, which means the new formulation get a more stable particle structure and allow solubilizing more DTX than the marketed one. According to the results of response surface methodology, we optimized the conditions to prepare DTX-loaded Polysorbate 80/Phospholipid mixed micelles and revealed the efficient encapsulation of DTX in the mixed micelles by using the self-assembly method [15]. The acquired DTX is formulated in a highly concentrated mixed solution containing Polysorbate 80, Phospholipid, and dehydrated alcohol. With 5% glucose solution, this concentrated mixed solution can be directly diluted to any precise concentration of DTX according to the clinical practice requirement. As well known, DTX filled in Polysorbate 80 micelles (Taxotere^®^) will precipitate within few hours, while the stability time of DTX-loaded Polysorbate 80/Phospholipid mixed micelles we prepared is more than 3 days. In the current study, we investigated the characteristics of the DTX-loaded Polysorbate 80/Phospholipid mixed micelles further, and the real value based on research of the blood compatibility, the pharmacokinetics, and the tissue distribution *in vivo *(*i.v*.) compared with DTX-loaded Polysorbate 80 micelles, which we made it simultaneously according to the preparation of Taxotere^® ^for a better reference (see http://www.taxotere.com and http://www.medsafe.govt.nz) [16].

As well known, the single component micelles is composed of amphipathic micromolecules, so the particle size of the single component micelles with drug filled in is usually far smaller than most of the drug loaded carriers [17]. In our study, the mean diameter of DTX-loaded Polysorbate 80 micelles was only 7.89 ± 1.97 nm. This is very important when the vesicles need to avoid the recognition of RES [18].

Although DTX-loaded Polysorbate 80/Phospholipid mixed micelles achieved the mean diameter of 13.89 ± 3.52 nm, which is still safely smaller than most of the drug vesicles. In addition, the TEM picture showed that the mixed micelles had a rod-like shape. It is different from the sphere morphology of liposomes prepared with Phospholipids. By close observation of the picture, bright and dark regions were observed in these mixed micelles further. The bright region might be attributed to the hydrophilic micelle shell, and the dark region might respond to the hydrophobic core of micelle with DTX. This core-shell structure of mixed micelles plays an important role in avoiding RES and providing long circulation time in blood [19].

The bioanalysis methods of DTX have been performed using HPLC with ultraviolet (UV) or mass spectrometric (MS) detection, or by enzyme-linked immunosorbent assays [20]. The lowest quantitation limits of these techniques were 10, 0.2, and 0.3 ng/mL, respectively. Since chromatographic methods are, in general, more selective and may provide information on drug metabolism, HPLC is usually preferred to immunoassays. Although MS detection is by far superior to UV detection, this technique is at the disposal of few laboratories or hospitals only, due to the high costs of the required equipment. For these reasons, the analysis method of DTX by means of HPLC with UV detection is generally considered a first choice for pharmacokinetic and tissue distribution studies in this work. According to the previous reports, a relatively good resolution was achieved for DTX by use of a manual pre-processing solid-phase extraction (SPE) procedure [21]. Herein, we developed a simpler reverse-phase HPLC analysis method of DTX concentration in plasma by use of a simpler liquid-liquid extraction instead of a SPE procedure, further with a HPLC mobile phase polarity regulator of tetrahydrofuran, and a HPLC mobile phase pH value slight regulator of acetic acid for a better optimization separation of DTX. Using this system, the retention time for DTX in rats plasma and mice tissue were approximately 10.7 and 11.2 min, respectively, with good resolution and without any interference from endogenous plasma constituents or DTX metabolites at these retention times. The total run time needed is only 13 min.

The pharmacokinetic profiles of the DTX encapsulated in the Polysorbate 80 micelles and Polysorbate 80/Phospholipid mixed micelles were consistent with a three-compartment pharmacokinetic model. The initial rapid decline represents distribution to the peripheral compartments and the late (terminal) phase is due, in part, to a relatively slow efflux of DTX from the peripheral compartment. These were agreeing with the previous research [22]. In addition, the results of hemolysis test and DTX plasma protein binding test showed that there is no obvious change to the blood distribution procedure of DTX captured in these micelles. All of these indicated that the encapsulation of DTX in Polysorbate 80/Phospholipid mixed micelles had not led to a change of pharmacokinetics.

To our knowledge, very little information has been published about the tissue distribution of DTX. In this work, the higher concentrations of DTX following intravenous administration in mice were found in the kidney, lung, and spleen than in plasma. The DTX concentration in the brain was very low, many brain samples could not be accurately measured because the concentration was lower than the limit of quantitation. These were consistent with the work reported by Zhao et al. [23]. Both the ovaries and uterus are too small to determine individually, so in this study, we placed them together. An interesting result in this work was that Polysorbate 80/Phospholipid mixed micelles increased the distribution of DTX in the ovaries and uterus, kidney, spleen, liver, and heart significantly, especially in liver. This show that DTX filled into the core of the mixed micelles get a preferred distribution in the organs rich in blood [24], not in plasma, this may due to the superior cell membrane biocompatibility of Phospholipid molecule. Because DTX had been proved effective in the treatment of tumor in these organs, therefore these results may improve the feasibility of reducing the dosage and intensifying the tumor tissues targeting ability of DTX. In addition, in case being encapsulated in Polysorbate 80 single component micelles or Polysorbate 80/Phospholipid mixed micelles, there is no evidence show that DTX have the preferential accumulation in RES tissues (spleen, lung, and liver etc.) compared with other tissues.

## Conclusion

A mixed micelle drug delivery system based on Polysorbate 80 and Phospholipid was developed and characterized as an effective alternative to DTX clinical preparation (Taxotere^®^). Our results demonstrated that the mixed micelles might efficiently load, protect, and retain DTX in biological environment. As a result, incorporating DTX in Polysorbate 80/Phospholipid mixed micelles can increase the drug residence amount in kidney, spleen, ovary and uterus, heart, and liver, and at the same time keep more consistent pharmacokinetics compared with Polysorbate 80 single component micelles (Taxotere^®^). These results suggested that Polysorbate 80/Phospholipid mixed micelle formulation can provide useful alternative dosage forms for clinical intravenous administration of DTX, and appear to be the best possible approach, which could bypass the limitation of current delivery system and provide a desirable therapeutic efficacy of DTX. A study on the formative mechanism of this drug loaded mixed micelles is under way in our lab.

## Abbreviations

CMC: critical micelle concentration; DTX: Docetaxel; HPLC: high-performance liquid chromatography; MS: mass spectrometric; OD: optical density; PDI: polydispersity coefficient; RES: reticuloendothelial system; SD: Sprague-Dawley; SPE: solid-phase extraction; TEM: transmission electron microscope; UV: ultraviolet.

## Competing interests

The authors declare that they have no competing interests.

## Authors' contributions

HS finished preparation of Polysorbate 80/Phospholipid mixed micellar, JR and KW finished characterization of prepared micellar, HG and CB finished the cell experiment, JW and XP helped HS to finish the animal experiment, XZ helped HS to analyze experimental data, DC designed the whole experiment and revised the manuscript.
